# The Structure of Bit-String Similarity Networks

**DOI:** 10.3390/e27010057

**Published:** 2025-01-10

**Authors:** David M. Schneider, Damián H. Zanette

**Affiliations:** 1 Centro Atómico Bariloche and Instituto Balseiro, Comisión Nacional de Energía Atómica, Universidad Nacional de Cuyo, Av. E. Bustillo 9500, San Carlos de Bariloche 8400, Argentina; davidmarsc@gmail.com; 2 Consejo Nacional de Investigaciones Científicas y Técnicas, Argentina

**Keywords:** bit-string models, similarity networks, structural properties

## Abstract

We study the structural properties of networks formed by random sets of bit strings—namely the ordered arrays of binary variables representing, for instance, genetic information or cultural profiles. Two bit strings are connected by a network link when they are sufficiently similar to each other, i.e., when their Hamming distance is below a certain threshold. Using both analytical and numerical techniques, we determine the degree distribution and the conditions for the existence of a giant component in this kind of network. In addition, we analyze their clustering, assortativity, and mean geodesic distance. We show that these properties combine features specific to random networks with characteristics that derive from the Hamming metrics implicit in the definition of similarity between bit strings.

## 1. Introduction

In recent decades, various stylized models of biological evolution—capturing in a simplified manner the elementary genetic mechanisms that drive evolutionary population dynamics, lineage diversification, speciation, and extinction—have been proposed [1,2,3,4,5]. To ease analytical and computational procedures, these models usually represent genomes as binary arrays, or bit strings (BSs), instead of the four-base nucleotide chains that constitute real genetic material. A BS is an ordered array of binary variables, with a given length *B*. It can be specified as a *B*-dimensional vector,

(1) $$b=(b_{1},b_{2},\ldots ,b_{B}),$$

where each component adopts one of two possible values, for instance, $b_{i}=0$ or 1 for i=1,2,…,B. This representation for genomes has become a standard tool in the theoretical study of viral RNA evolution [6]. BSs have also been used as the building blocks of models for complex systems of various kinds. For example, Schulze’s model of language evolution [7,8] and its variations [9] characterize a language by a collection of dichotomic properties, which naturally calls for a representation in terms of binary variables. Similarly, inspired by Axelrod’s model of culture dissemination [10], recent models of culture evolution conceive cultural profiles as sets of binary features represented by BSs [11]. This kind of dichotomic categorization underlies the binary classification of digitized information, a central task in automated data analysis [12].

In many of the evolutionary models referred to above, dynamical rules prescribe that any interaction between two elements is only allowed when the elements are comparable to some degree. For instance, two organisms can become involved in a reproductive event only if they are “compatible”, namely if their genomes are sufficiently similar to each other [1,4]. Likewise, cultural interaction is only possible between individuals who already possess a certain degree of cultural similarity [10]. The comparison between two BSs of the same length *B*, say b and $b^{\prime }$, is usually performed by calculating their Hamming distance,

(2) $$H\left(b,b^{\prime }\right)=\sum_{i=1}^{B}\left|b_{i}-b_{i}^{\prime }\right|,$$

which equals the number of bits where b and $b^{\prime }$ differ. In fact, *H* is an integer number with 0≤H≤B. Any interaction between two elements in the above models becomes possible when the Hamming distance of the corresponding BSs is below a prescribed threshold.

**Figure 1:**
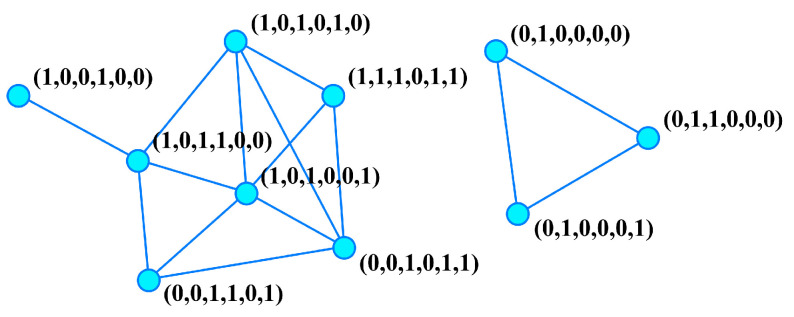
A small bit-string similarity network of size N=10, with BS length B=6, and threshold G=2.

In this contribution, we consider a class of networks formed by a set of BSs that are connected depending on their degree of similarity. Specifically, we take *N* mutually different, randomly chosen BSs of length *B* and establish links joining each pair when their Hamming distance is lower than or equal to a threshold *G*, i.e., H≤G, with 1≤G≤B. Figure 1 shows an example of these BS similarity networks with small values of *N*, *B*, and *G*. In the models described in the preceding paragraphs, where elements represented by BSs interact with each other when their Hamming distance is sufficiently small, this construction yields the corresponding interaction network.

In the following sections, first, we show that random sets of BSs, as those used to build our networks, emerge spontaneously as the result of neutral evolution in populations of constant size. Then, we present analytical and numerical results on the global structural properties of BS similarity networks. We study the degree distribution, the appearance of a giant component, clustering, assortativity, and mean geodesic distance, as functions of *N*, *B*, and *G*. We show that these properties combine the features typically found in both random and non-random networks. Our results are summarized in the last section.

## 2. Random Bit-String Sets as the Outcome of Neutral Evolution

Before studying the structure of the BS similarity networks defined in the Introduction, it is opportune to show that the sets of BSs on which these networks are built arise spontaneously as the result of simple evolutionary mechanisms based on random mutations and in the absence of selection pressure—i.e., under neutral evolution [13]. Specifically, we consider a population of *N* BSs of length *B* that are initially identical, for instance, $b_{n}=\left(0,0,\ldots ,0\right)$ for all n=1,2,…,N. At each time step, a “parent” BS $b_{\mathrm{parent}}$ is chosen at random, and a “child” BS $b_{\mathrm{child}}$ is created by mutating $b_{\mathrm{parent}}$. The mutation amounts to switching a single, randomly chosen bit in $b_{\mathrm{parent}}$ to its opposite value, either 0→1 or 1→0. Then, another randomly selected BS “dies” and is replaced by $b_{\mathrm{child}}$. In this toy evolutionary model, thus, increasingly dissimilar BSs are created, while the population size remains constant.

A convenient way of characterizing the composition of a given set of BSs—in particular, for comparison with a random set—is to determine the distribution of Hamming distances between all its elements. In a set of random BSs of length *B*, the expected frequency of a Hamming distance *H* equals the probability that two random BSs differ by exactly *H* bits, namely

(3) fHrandom=2−BBH,

for H=0,1,…,B. In a numerical realization of our evolutionary model, we measure the frequency of each value of the Hamming distance, $f_{H}\left(T\right)$, as the number of time steps *T* increases, and compare with $f_{H}^{\mathrm{random}}$ by computing the difference

(4) $$D\left(T\right)=\sqrt{\sum_{H=0}^{B}{\left[f_{H}\left(T\right)-f_{H}^{\mathrm{random}}\right]}^{2}}$$

between both distributions.

**Figure 2:**
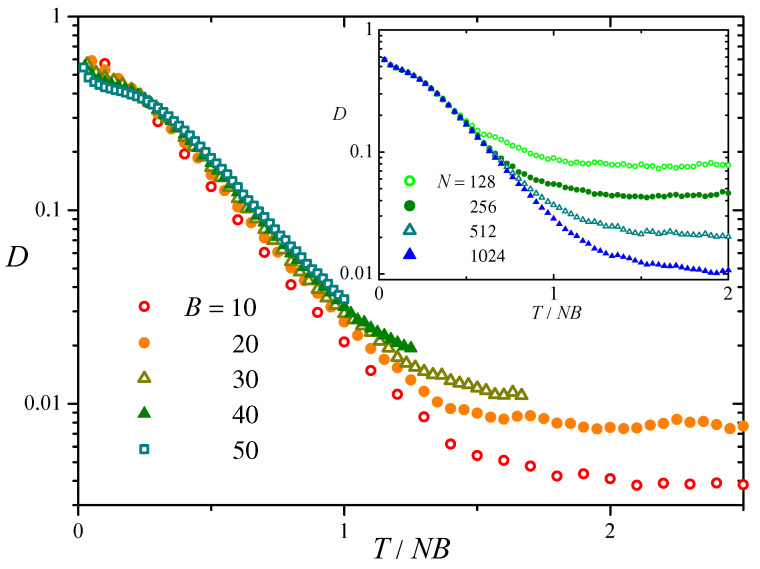
Main panel: Time evolution of the difference *D* between the distribution of Hamming distances in the evolutionary model described in the text and in a random set of bit strings, Equation (4), for N=1024 and five values of *B*. In the horizontal axis, the number of steps *T* is normalized by the product NB. For each *B*, the results are averaged over 50 realizations of the model. Inset: As in the main panel, for B=30 and four values of *N*.

The main panel of Figure 2 shows our measurements of D(T) plotted versus the rescaled time T/NB, for N=1024 and five values of *B*. For each *B*, results correspond to an average over 50 realizations of the evolution. The inset shows similar results for B=30 and four values of *N*. In both plots, a unit in the horizontal scale corresponds to the average waiting time for any of the NB bits in the population to be switched by mutation. We see that, after a short transient, D(T) reaches a regime of exponential decay, decreasing with a rate that is essentially independent of *N* and *B*, at least, for large *B*. Consequently, as may have been expected, the typical number of mutations needed for D(T) to decrease by a given factor in this exponential regime is proportional to both *N* and *B*.

For longer times, the difference D(T) ceases to decrease and varies irregularly around a constant value. This asymptotic behavior can be ascribed to the remnant fluctuations of $f_{H}\left(T\right)$ around the expected value $f_{H}^{\mathrm{random}}$. As seen in Figure 2, the asymptotic value of *D* increases with *B*, as expected from the fact that the calculation of *D*, Equation (4), involves a sum of B+1 terms. On the other hand, it decreases with the population size as $N^{-1}$. This dependence on *N* can be understood by taking into account that, by virtue of the law of large numbers, the long-time fluctuations of $f_{H}\left(T\right)$ around $f_{H}^{\mathrm{random}}$ are expected to be of order $Z^{-1/2}$, where *Z* is the number of data used to evaluate $f_{H}\left(T\right)$. In our case, *Z* is the number of pairs of BSs in the population, over which we calculate the Hamming distances, so that $Z\propto N^{2}$. This implies that $D∼|f_{H}\left(T\right)-f_{H}^{\mathrm{random}}|∼N^{-1}$. Thus, up to stochastic fluctuations due to the finite size of the population, the distribution of Hamming distances between BSs converges with time to that of a random set of the same size.

Similar qualitative behavior is observed in a few variants of the above evolutionary model. For instance, if, instead of replacing a randomly chosen BS, $b_{\mathrm{child}}$ replaces its own parent $b_{\mathrm{parent}}$, a random set of BSs is obtained at long times just by construction. We have also verified that D(T) decays qualitatively in the same manner as shown in Figure 2, when bit mutations are combined with recombination, where a child is created by concatenating two complementary portions taken from different parents [4].

## 3. Structural Properties

In this section, we study the global structural properties of BS similarity networks. As shown below, some of them can be evaluated analytically while others require resorting to numerical computation. In qualitative terms, our main conclusion is that networks in this class combine the characteristic features of random graphs, such as the degree distribution and the appearance of a giant component, with properties that cannot be explained as the result of aleatory connections, such as their clustering.

### 3.1. Degree Distribution, the Giant Component, and Richness

First, we calculate the degree distribution $g_{k}$ of our networks, namely the probability that a node has exactly *k* neighbors. Among the $M=2^{B}$ possible BSs of length *B*, there are BH at a Hamming distance *H* from any of them (say, b). Therefore, the total number of BSs at a distance less than or equal to *G* from b, excluding b itself, is

(5) MG=∑H=1GBH.

The fraction of BSs at a distance *H* from b, such that 1≤H≤G, is

(6) $$\mu =\frac{M_{G}}{M-1}.$$

The results presented in the following show that μ is a natural parameter to characterize the connectivity properties of BS similarity networks. Note, from Equations (5) and (6), that μ adopts discrete increasing values as the threshold *G* varies from 1 to *B*.

Our networks are built by choosing at random *N* of the *M* possible BSs. The probability that *k* of them are among the $M_{G}$ at distance *H* from b, with 1≤H≤G, is

(7) gk=N−1kμk(1−μ)N−1−k,

for k=0,1,…,N−1. We have numerically verified that, up to a high precision, this is indeed the degree distribution of BS similarity networks of size *N* and maximal Hamming distance *G*. Note that it coincides with the degree distribution of an Erdős–Rényi random network with *N* nodes and μN(N−1)/2 links [14]. The mean number of links per node is 〈k〉≡z=μ(N−1).

Assuming that it is valid to apply the theory of random networks [15] to our case, we can calculate the critical value of μ, which we denote as $\mu_{c}$, above which a giant component exists in the network. We recall that a giant component is strictly well defined for infinitely large networks, as a connected component that comprises a finite part of the network. In finite, but sufficiently large networks, it is associated with the largest connected component [14]. To evaluate $\mu_{c}$, we start by computing the generating function of the degree distribution $g_{k}$:

(8) $$\Gamma \left(x\right)=\sum_{k=0}^{N-1}g_{k}x^{k}={\left(1-\mu +\mu x\right)}^{N-1}.$$

The critical point $\mu_{c}$ is the minimum value of μ for which the equation

(9) $$u=\frac{\Gamma^{\prime }\left(u\right)}{\Gamma^{\prime }\left(1\right)}={\left(1-\mu +\mu u\right)}^{N-2}$$

has a solution u<1 [15], namely

(10) $$\mu_{c}=\frac{1}{N-2}.$$

For $\mu \geq \mu_{c}$, the fraction of the network in the giant component is

(11) $$S=1-u^{\left(N-1)/(N-2\right)},$$

where *u* is the solution to Equation (9). For N≫1, Equations (9) and (11) can be approximated as

(12) $$u=\exp\left[-\frac{\mu }{\mu_{c}}\left(1-u\right)\right],\;\;\;\;\;\;\mathrm{and}\;\;\;\;\;S=1-u,$$

respectively. For large networks, therefore, it is clear that the relative size of the giant component depends on *B*, *G*, and *N* through the ratio $\mu /\mu_{c}$ only. Note that, when *N* is large, $\mu /\mu_{c}$ is a direct measure of the average number of neighbors per site:

(13) $$z=\mu \left(N-1\right)=\frac{\mu }{\mu_{c}}\frac{N-1}{N-2}\approx \frac{\mu }{\mu_{c}}.$$

**Figure 3:**
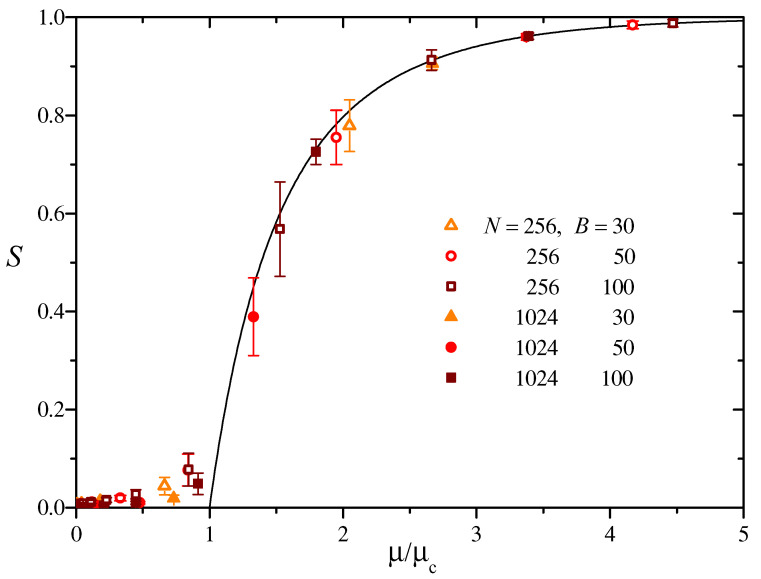
Full curve: The solution to Equation (12) for the fraction of the network inside the giant component, *S*, as a function of the ratio $\mu /\mu_{c}$. Symbols: Numerical measurement of the relative size of the largest component for various combinations of *N* and *B*, averaged over 50 realizations for each parameter set. Error bars represent the standard deviation over realizations.

The curve in Figure 3 is the solution to Equation (12) for the relative size of the giant component, *S*, as a function of $\mu /\mu_{c}$. Symbols, in turn, show numerical results for the fraction of the network in the largest component, for two values of *N*, and three values of *B*. Error bars represent the standard deviation over sets of 50 realizations for each parameter set. As expected, this dispersion is the largest in the transition zone, $\mu \approx \mu_{c}$, and becomes negligible elsewhere. Apart from typical finite-size effects for $\mu ≲\mu_{c}$, the agreement with the analytical prediction is excellent, indicating that the theory of random networks correctly describes the appearance and growth of the giant component in BS similarity networks.

**Figure 4:**
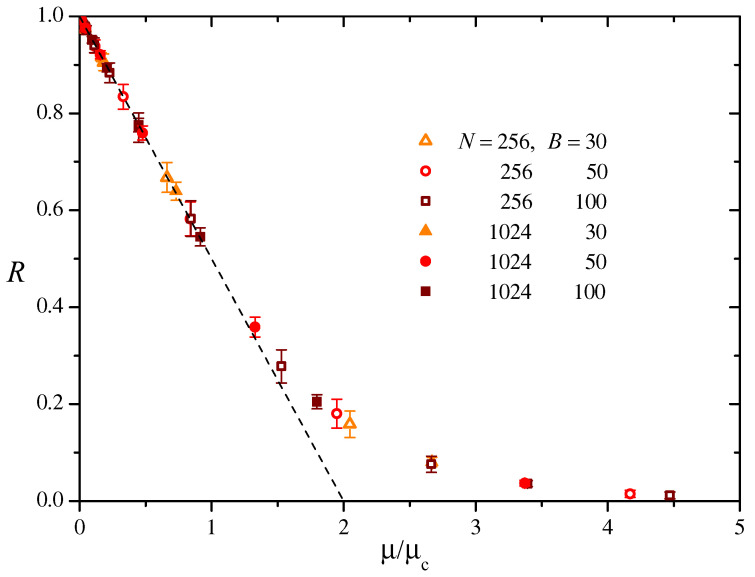
The richness *R*, given by the number of connected components in the network divided by the network size *N*, as a function of $\mu /\mu_{c}$, estimated from the same numerical realizations as in Figure 3. Error bars represent the standard deviation over realizations. The dashed straight line is the analytical approximation for small $\mu /\mu_{c}$ discussed in the text, Equation (14).

An accessory quantity of interest for the evolutionary models quoted in the Introduction is the *richness R*, namely the ratio between the number of connected components in the network and its size *N*. The richness is a measure of diversity in the population, since each connected component is associated with a different “species” [4,5]. Figure 4 shows our numerical estimation for the richness corresponding to the same realizations as in Figure 3. Note that the collapse of results for different values of *B* and *N* onto a single curve, indicating that—at least within this parameter range—*R* depends on $\mu /\mu_{c}$ only, much as the relative size of the giant component *S*.

As expected, R≈1 as μ→0. In this limit, in fact, most nodes in the networks are disconnected from each other, and the number of connected components is close to *N*. For small $\mu /\mu_{c}$, the richness seems to decrease linearly. However, beginning at $\mu /\mu_{c}≳1$, where the giant component first appears and begins to grow, *R* deviates from the linear behavior to approach $N^{-1}$ for a large $\mu /\mu_{c}$, where the giant component comprises the whole network.

The linear decrease in *R* for $\mu /\mu_{c}≲1$ can be readily understood taking into account that, for sufficiently small μ, the network is formed by isolated nodes and, at most, tiny connected components. From Equation (7), the expected number of isolated nodes is $g_{0}N$ which, for a large *N*, is approximately given by $N\exp(-\mu /\mu_{c})$. This is the number of components of size one. Assuming that all the other nodes form connected components of size two, the number of these components would be $N[1-\exp\left(-\mu /\mu_{c}\right)]/2$. In this situation, thus, the richness would be given by the sum of the numbers of components of sizes one and two divided by *N*:

(14) $$R=\exp\left(-\mu /\mu_{c}\right)+\frac{1-\exp(-\mu /\mu_{c})}{2}\approx 1-\frac{\mu }{2\mu_{c}},$$

where the approximation holds for small $\mu /\mu_{c}$. This prediction is plotted in Figure 4 as a dashed line. We see that, actually, it gives a satisfactory explanation of the dependence of *R* up to $\mu /\mu_{c}\approx 1$.

### 3.2. Clustering

The statistical tendency of the neighbors of a given node in a network to be, in turn, mutual neighbors is called clustering or transitivity. This feature, especially ubiquitous in social networks [14,16,17], corresponds to an enhanced probability of finding groups of well-interconnected nodes with respect to the situation where links between nodes are distributed at random. In our BS similarity networks, clustering is expected to differ from that of random networks due to the structure induced by the Hamming metrics involved in their construction. Concretely, suppose that a given BS in the network has two neighbors at a Hamming distance H=1. Since the two neighbors are, by construction, not identical, their mutual Hamming distance is necessarily H=2. Consequently, if the threshold *G* is greater than or equal to two, the two neighbors will be mutually connected. Similarly, if the two neighbors are at distances H=1 and H=2 from the given BS, their mutual distance cannot be larger than H=3, and so on. The Hamming metrics thus favor the mutual connection between neighbors of any given BS, with the ensuing growth of clustering.

Several measures have been proposed to quantify clustering [17]. For the BS similarity networks, we here compute the *mean clustering coefficient C*, as originally defined for small-world networks [16]. The mean clustering coefficient is the average over all the nodes in the network of the *local* clustering coefficient, $C_{i}$, given by the ratio between the number of pairs of neighbors of node *i* which are in turn mutual neighbors, and the total number of pairs of neighbors of the same node. In an Erdős–Rényi random network with *N* nodes and an average of *z* neighbors per node, the mean clustering coefficient decreases with the network size as $C∼zN^{-1}$ [17].

To evaluate the mean clustering coefficient of BS similarity networks, we first consider a generic BS $b_{0}$ and two other BSs $b_{1}$ and $b_{2}$ at Hamming distances $H_{1}$ and $H_{2}$ from $b_{0}$, respectively. If the three BSs belong to a similarity network with threshold *G*, $b_{1}$ and $b_{2}$ will be neighbors of $b_{0}$ when $H_{1},H_{2}\leq G$. They will contribute to the local clustering of $b_{0}$ if they are in turn mutual neighbors or, in other words, if their mutual distance *g* is lower than or equal to *G*. Since the number of neighbors at any given distance is the same for all BSs, we can ease the calculations assuming that $b_{0}=\left(0,0,\ldots ,0\right)$. With this choice, $H_{1}$ and $H_{2}$ are the number of ones in $b_{1}$ and $b_{2}$, respectively.

Without generality loss, we take $H_{2}\geq H_{1}$. The distance *g* between $b_{1}$ and $b_{2}$ is the number of bits where the ones in $b_{1}$ do not coincide with those in $b_{2}$. The direct inspection of the possible distributions of ones along both BSs shows that the distance adopts the values $g=H_{1}+H_{2}-2h$, with $h=0,1,\ldots ,H_{1}$, where *h* is the number of bits where the ones do coincide. For a fixed value of *h*, the total number of pairs $\left(b_{1},b_{2}\right)$ whose mutual distance equals $H_{1}+H_{2}-2h$ is given by the product of (i) the number of BSs of length *B* with $H_{1}$ ones, times (ii) the number of ways of selecting *h* bits from $H_{1}$ bits with ones, times (iii) the number of ways of selecting $H_{2}-h$ bits from $B-H_{1}$ bits with zeros, namely

(15) πh=BH1H1hB−H1H2−h.

Thus, the probability that such a pair does occur in the network equals

(16) Πh=πh∑h′=0H1πh′=BH2−1H1hB−H1H2−h.

The probability that their mutual distance is lower than or equal to *G* is then

(17) P(H1,H2)=∑h=h0H1Πh=BH2−1∑h=h0H1H1hB−H1H2−h,

with $h_{0}=\max\left\{0,\left[\left(H_{1}+H_{2}-G+1\right)/2\right]\right\}$, where [·] indicates the integer part. It can be shown that the combination of binomial coefficients in Equations (16) and (17) is symmetric with respect to $H_{1}$ and $H_{2}$,

(18) BH2−1H1hB−H1H2−h=BH1−1H2hB−H2H1−h.

Equation (17) is therefore also valid when $H_{2}<H_{1}$, with the convention that the binomial coefficient ij vanishes whenever j>i.

Taking into account that the total number of BSs at a distance *H* from any one of them is BH [cf. Equation (5)], the mean clustering coefficient *C*, given by the average of local clustering all over the network, can be evaluated as the sum of the contributions coming from all distances $H_{1}$ and $H_{2}$ of all pairs of mutual neighbors at such distances:

(19) C=∑H1=1G∑H2=1GBH1BH2P(H1,H2)=∑H1=1G∑H2=1G∑h=h0H1B!h!(H1−h)!(H2−h)!(B−H1−H2+h)!,

with $h_{0}$ as in Equation (17). Note that this result is independent of the network size *N*, since it originates in purely probabilistic considerations. As such, it is expected to hold for large networks.

**Figure 5:**
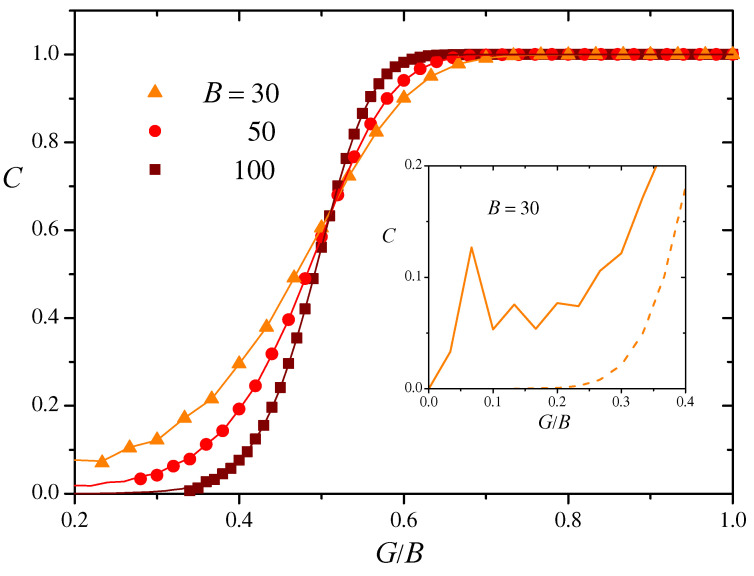
Main panel: Symbols show numerical estimates for the mean clustering coefficient *C* of BS similarity networks with N=1024 and three values of the bit-string length *B*, as a function of the ratio G/B, averaged over 50 realizations. The standard deviation of *C* over realizations is smaller than the size of symbols. Lines join the analytical values for *C* obtained from Equation (19). Inset: The full line shows *C* for B=30 and small values of the threshold *G*. The dashed line shows *C* for an Erdős–Rényi network with the same mean degree for each value of G/B.

Symbols in Figure 5 show the numerical results of the mean clustering coefficient *C* averaged over the 50 realizations of BS similarity networks of size N=1024 and three values of *B*, as a function of the ratio G/B. For each *B*, lines join the analytical values of *C* obtained from Equation (19) varying the threshold *G*. The agreement is excellent, which validates the above probabilistic arguments. Our numerical results for other network sizes, starting at N=128, also agree very well with the analytical prediction. As expected, clustering increases as *G* grows and the network connectivity becomes larger. The S-shaped dependence on *G* shows an inflection point in the zone where the giant component has already appeared. However, even below this zone, mean clustering adopts relatively large values, well above those expected for random networks. The full line in the inset of Figure 5 shows the analytical value of *C* for B=30 and small values of the threshold *G*. For comparison, the dashed line shows the mean clustering coefficient of an Erdős–Rényi random network with the same mean number of neighbors.

### 3.3. Degree Assortativity

Given an attribute *a* assigned to each node in a network, the *assortativity* with respect to *a* measures the statistical correlation between the values of *a* in pairs of connected nodes. Assortativity is defined as the Pearson correlation coefficient of *a* computed over all pairs of neighbors [14,17], and is therefore confined to the interval [−1,1]. A generic attribute that allows for the computation of assortativity is the number of neighbors of each node, namely the node degree *k*. Degree assortativity is large and positive when nodes with either many or few neighbors are preferentially interconnected, while it is large and negative when connections mainly occur between nodes with disparate degrees. On the other hand, assortativity is close to zero in networks with a random distribution of links, such as Erdős–Rényi networks.

We could not find an accurate analytical prediction for the assortativity of BS similarity networks. Our estimations are therefore based on numerical calculations. A convenient way of computing the Pearson correlation coefficient between the degrees of neighbor nodes is [14,18]

(20) $$A=\frac{4\langle k_{1}k_{2}\rangle -{\langle k_{1}+k_{2}\rangle }^{2}}{2\langle k_{1}^{2}+k_{2}^{2}\rangle -{\langle k_{1}+k_{2}\rangle }^{2}},$$

where 〈·〉 indicates the average over all the links in the network, with $k_{1}$ and $k_{2}$ being the degrees of the two nodes at the ends of each link. In our numerical calculations, we have used this expression to compute the assortativity.

**Figure 6:**
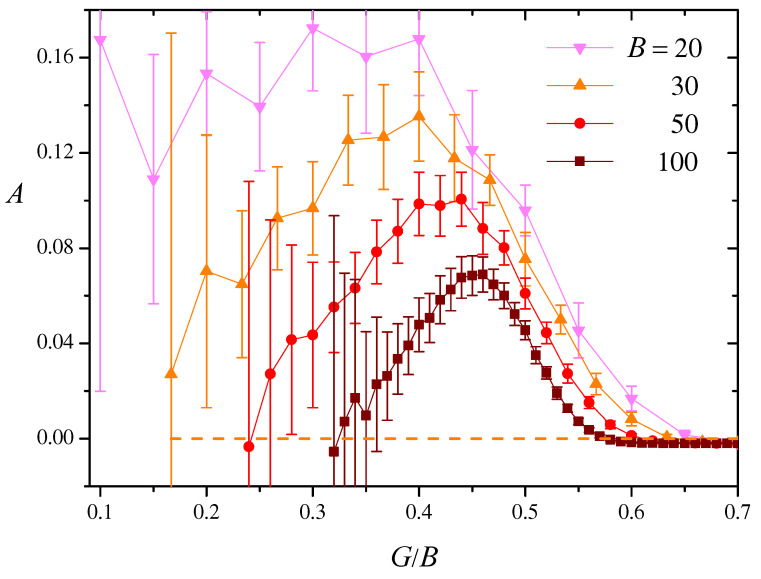
The assortativity *A*, computed as in Equation (20) as a function of G/B, for a series of 50 realizations with N=1024 and four values of *B*. Symbols and error bars, respectively, represent the averages and standard deviations over each series of realizations. The dashed line shows numerical results for *A* in Erdős–Rényi networks of size N=1024 which, for each value of G/B, have the same mean degree as the BS similarity networks with B=30.

We computed *A* in a series of 50 realizations of BS similarity networks for various combinations of the sizes *N* and BS lengths *B*, as a function of the threshold *G*. Our first finding is that, as much as the mean clustering coefficient *C* studied in the preceding section, *A* becomes independent of *N* for large network sizes. This is not unexpected, since assortativity is a structural feature defined in terms of statistical properties, as is *C*. Figure 6 shows our numerical results for N=1024 and four values of *B*. Symbols and error bars, respectively, represent averages and standard deviations over each series of realizations. We see that, in all cases, the average assortativity reaches small positive values, A≲0.17, with typically large dispersions, which can be much larger than the average itself. As a general trend, *A* decreases as the BS length *B* grows. For each value of *B*, it reaches a maximum at G/B<0.5. Beyond the maximum, it shows a rapid decrease towards zero as the network connectivity grows.

The dashed line in Figure 6 shows the assortativity *A* computed numerically for Erdős–Rényi networks of size N=1024, with the same mean degree as BS similarity networks with B=30. Its value cannot be discerned from zero within the plot scale. We see that, as compared with their random counterparts, the assortativity of BS similarity networks reaches statistically significant levels. However, the overall small values of *A* suggest that assortativity is not a particularly relevant structural feature of our networks.

### 3.4. Mean Geodesic Distance

Lastly, we studied the mean geodesic distance in BS similarity networks. The geodesic (or chemical) distance between two nodes in a network is given by the number of links along the shortest path joining the two nodes with each other [14,17]. Naturally, this quantity is well defined when such a path exists, which requires that the two nodes belong to the same connected component. By convention, when the two nodes are in different components, their geodesic distance is taken to be infinite. The *mean geodesic distance L*, therefore, is usually defined as the arithmetic average of geodesic distances over all pairs where the two nodes belong to the same component, i.e., with finite geodesic distance. It is a direct measure of network connectivity, and is related to robustness and percolation properties [19,20].

**Figure 7:**
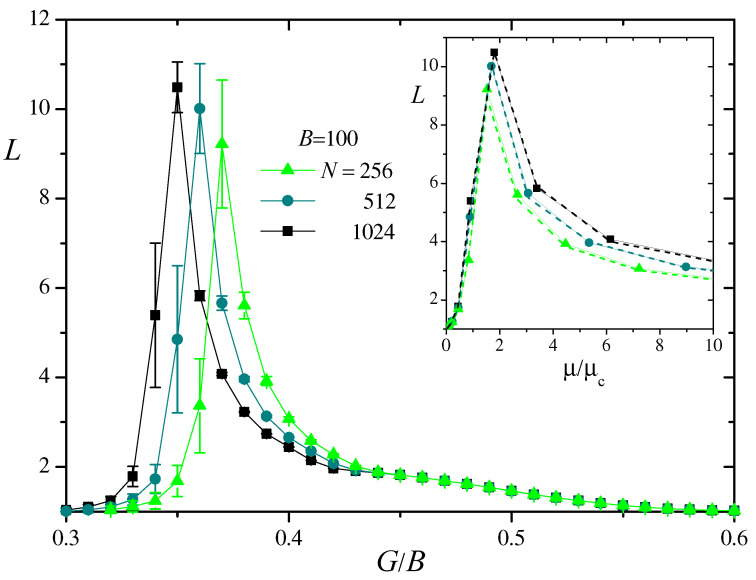
Main panel: Numerical results for the mean geodesic distance *L* in BS similarity networks with B=100 and three values of *N*, as a function of G/B. Symbols are averages over 50 realizations and error bars show the corresponding standard deviations. Lines joining symbols are added for clarity. Inset: The same results for *L* as a function of $\mu /\mu_{c}$ in the zone of the maximum. For clarity, error bars have been omitted. Dashed lines join numerical results for *L* in Erdős–Rényi random networks of the same size and for the same mean degree.

The main panel of Figure 7 shows our numerical measurements of *L* for BS similarity networks with B=100 and three values of *N* as a function of G/B. Symbols stand for averages over 50 realizations and error bars are the corresponding standard deviations. Results for other values of *B* show the same qualitative behavior. For a small G/B, when the network is poorly connected, we find L≈1. In this limit, in fact, each node is connected with at most one neighbor, at a unitary geodesic distance. The same value of *L* is obtained when G/B is sufficiently large, with virtually all nodes connected to each other. Both for small and large G/B, fluctuations of *L* between realizations are negligible.

In the intermediate zone, in contrast, *L* shows a sharp peak, where it reaches significantly larger average values and fluctuations. The height of the peak increases slowly with *N*. The inset of Figure 7 shows the same numerical results plotted versus the ratio $\mu /\mu_{c}$, in the zone of the maximum of *L*. We see that the peak is located in the interval $1≲\mu /\mu_{c}≲2$, i.e., just above the appearance of the giant component. In this zone, where several connected components of different sizes are still present and the giant component is just beginning to grow (cf. Figure 3 and Figure 4), the overall network structure is most irregular and geodesic distances can reach large values.

It turns out, however, that this is exactly the same behavior as observed for the mean geodesic distance in random networks. Dashed lines in the inset of Figure 7 join numerical results for *L* measured in Erdős–Rényi networks of the same size *N* and the same mean number of neighbors *z* as the corresponding BS similarity networks, averaged over 50 realizations. Results for both kinds of network are indistinguishable from each other within numerical fluctuations. We conclude that the specific structure of BS similarity networks has no impact on their mean geodesic distance, which only reveals the underlying random choice of the BSs used to build the network.

## 4. Conclusions

In this paper, we have studied a class of complex networks whose nodes are bit strings—namely, ordered arrays of binary variables—of fixed length *B*. The set of *N* bit strings present in the network is chosen at random among the $2^{B}$ possibilities. Network links join sufficiently similar bit strings, specifically, those whose Hamming distances are at most equal to a given threshold *G*. As we have discussed in the Introduction, these bit-string similarity networks coincide with the interaction patterns in a variety of evolutionary models where bit strings represent individual genomes and interactions are reserved to pairs of genetically comparable individuals. We have shown in Section 2 that the random sets of bit strings on which our networks are built arise spontaneously under the action of neutral evolution under various evolutionary mechanisms.

Our main results, reported in Section 3, refer to global structural properties of bit-string similarity networks, which we studied as functions of the parameters *N*, *B*, and *G*. We have obtained exact analytical results for the degree distribution, the appearance of a giant component—which, as in many other network classes, has the character of a critical phenomenon—and the mean clustering coefficient. Meanwhile, the number of connected components, the assortativity, and the mean geodesic distance, have been studied numerically.

In qualitative terms, the most interesting property detected in our bit-string similarity networks is a combination of features which are characteristic of random networks with other properties which disclose non-trivial correlations in their structure. For instance, with a suitable identification of parameters, their degree distribution exactly coincides with that of Erdős–Rényi random networks. Likewise, the appearance of a giant component can be accurately predicted using the generating function formalism for networks with prescribed degree distribution and randomly distributed links [15]. According to numerical results, the mean geodesic distance of bit-string similarity networks also coincides to a large precision with the same quantity measured for their Erdős–Rényi counterparts. On the contrary, clustering and, to a lesser extent, assortativity, reach significantly high levels, in contrast with their negligible values in large random networks [14]. This merging of random and non-random features can be ascribed to the combined impact of the two main ingredients that take part in the construction of bit-string similarity networks. On the one hand, the random choice of the set of bit strings present in each realization of the network determines that the neighbors of a given node will also be a random subset of all the possible bit strings of a given length. On the other, as exemplified at the beginning of Section 3.2, the Hamming metrics implicit in the way nodes are connected establish a statistical interdependence between their neighborhoods. This affects clustering and assortativity which are, precisely, different measures of such correlations. We stress that the joint occurrence of random and non-random attributes is typical of other complex structures, notably, small-world networks, which combine short geodesic distances with large clustering [17]. This feature has already been pointed out in the real-life networks of various origins [16]. It remains to be assessed whether other specific structural properties of bit-string similarity networks are found in the interaction patterns of actual biological and sociocultural systems.

Relaxing the binary nature of the ordered arrays used to build the networks studied here would allow for a more realistic representation of genome chains or, generally, more complex characterizations of individual features. For arrays where each variable can adopt more than two states, or even its own set of states, the Hamming distance can still be defined as the number of variables where two arrays differ from each other. Since the properties of the Hamming metrics are essentially the same as for bit strings, we expect similar structural properties in the resulting similarity networks. We also point out that the Hamming distance naturally provides a way to define a weighted network—a variant not explored in the present contribution—assigning, for example, larger weights to the links between bit strings at shorter distances. Finally, an interesting extension related to evolutionary models would be to study the similarity networks formed by collections of bit strings deriving from evolutionary mechanisms other than neutral evolution, under the action of selection pressure. All these variants may stimulate future work on dynamical processes occurring on such structures, modeling both biological and social phenomena.

## Data Availability

The original contributions presented in this study are included in the article. Further inquiries can be directed to the corresponding author.

## References

[1] Higgs P.G., Derrida B. (1991). Stochastic models for species formation in evolving populations. J. Phys. A Math. Gen..

[2] Penna T. (1995). A bit-string model for biological aging. J. Stat. Phys..

[3] Falkiewicz D.M., Makowiec D. (2021). Bit-string model of biological speciation: Revisited. Phys. A.

[4] de Aguiar M.A.M., Baranger M., Baptestini E.M., Kaufman L., Bar-Yam Y. (2009). Global patterns of speciation and diversity. Nature.

[5] Schneider D., Baptestini E.M., de Aguiar M.A.M. (2016). Diploid versus haploid models of neutral speciation. J. Biol. Phys..

[6] Elena S.F., Solé R.V., Sardanyés J. (2010). Simple genomes, complex interactions: Epistasis in RNA virus. Chaos Interdiscip. J. Nonlinear Sci..

[7] Schulze C., Stauffer D. (2005). Monte Carlo simulation of the rise and the fall of languages. Int. J. Mod. Phys. C.

[8] Zanette D.H. (2008). Analytical approach to bit-string models of language evolution. Int. J. Mod. Phys. C.

[9] de Olivera P.M.C., Stauffer D., Lima F.W.S., Sousa A.O., Schulze C., Moss de Oliveira S. (2007). Bit-strings and other modifications of Viviane model for language competition. Phys. A.

[10] Axelrod R. (1997). The dissemination of culture: A model with local convergence and global polarization. J. Confl. Resolut..

[11] Pascual I., Aguirre J., Manrubia S., Cuesta J.A. (2020). Epistasis between cultural traits causes paradigm shifts in cultural evolution. R. Soc. Open Sci..

[12] Flach P. (2012). Machine Learning. The Art and Science of Algorithms that Make Sense of Data.

[13] Duret L. (2008). Neutral theory: The null hypothesis of molecular evolution. Nat. Educ..

[14] Newman M.E.J. (2010). Networks: An Introduction.

[15] Newman M.E.J., Strogatz S.H., Watts D.J. (2001). Random graphs with arbitrary degree distributions and their applications. Phys. Rev. E.

[16] Watts D.J., Strogatz S.H. (1998). Colletive dynamics of ‘small-world’ networks. Nature.

[17] Newman M.E.J., Barabási A.L., Watts D.J. (2006). The Structure and Dynamics of Networks.

[18] Barrenas F., Chavali S., Holme P., Mobini R., Benson M. (2009). Network properties of complex human disease genes identified through genome-wide association studies. PLoS ONE.

[19] Cohen R., Erez K., ben-Avraham D., Havlin S. (2000). Resilience of the internet to random breakdowns. Phys. Rev. Lett..

[20] Callaway D.S., Newman M.E.J., Strogatz S.H., Watts D.J. (2000). Network robustness and fragility: Percolation on random graphs. Phys. Rev. Lett..

